# 6Questionnaire-based approach to assess schoolchildren's physical fitness and its potential role in exploring the putative impact of helminth and *Plasmodium *spp. infections in Côte d'Ivoire

**DOI:** 10.1186/1756-3305-4-116

**Published:** 2011-06-24

**Authors:** Thomas Fürst, Ivan Müller, Jean T Coulibaly, Adrien K Yao, Jürg Utzinger, Eliézer K N'Goran

**Affiliations:** 1 Department of Epidemiology and Public Health, Swiss Tropical and Public Health Institute, Basel, Switzerland; 2 University of Basel, Basel, Switzerland; 3 Department of Public Health, Institute for Sports and Sports Sciences, University of Basel, Basel, Switzerland; 4 Centre Suisse de Recherches Scientifiques en Côte d'Ivoire, Abidjan, Côte d'Ivoire; 5 UFR Biosciences, Université de Cocody-Abidjan, Abidjan, Côte d'Ivoire; 6 Services de Santé Scolaire et Universitaire, Agboville, Côte d'Ivoire

## Abstract

**Background:**

Disability weights (DWs) are important for estimating burden of disease in terms of disability-adjusted life years. The previous practice of eliciting DWs by expert opinion has been challenged. More recent approaches employed quality of life (QoL) questionnaires to establish patient-based DWs, but results are ambiguous.

**Methods:**

In early 2010, we administered a questionnaire pertaining to physical fitness to 200 schoolchildren in Côte d'Ivoire. Helminth and *Plasmodium *spp. infections were determined and schoolchildren's physical fitness objectively measured in a maximal multistage 20 m shuttle run test. Associations between objectively measured and self-reported physical fitness and between self-reported physical fitness and infection status were determined. Spearman rank correlation coefficient, uni- and multivariable linear regression models adjusting for children's age and sex, ambient air temperature and humidity, Fisher's test, χ² and t-test statistics were used for statistical analysis.

**Results:**

The prevalence of *Schistosoma haematobium, Plasmodium *spp., *Schistosoma mansoni*, hookworm and *Ascaris lumbricoides *in 167 children with complete parasitological results was 84.4%, 74.9%, 54.5%, 14.4% and 1.2%, respectively. High infection intensities and multiple species parasite infections were common. In the 137 children with complete data also from the shuttle run test, we found statistically significant correlations between objectively measured and self-reported physical fitness. However, no statistically significant correlation between the children's parasitic infection status and self-reported physical fitness was identified. An attrition analysis revealed considerably lower self-reported physical fitness scores of parasitized children who were excluded from shuttle run testing due to medical concerns in comparison to parasitized children who were able to successfully complete the shuttle run test.

**Conclusions:**

Our QoL questionnaire proofed valid to assess children's physical fitness in the current study area. Reasons why no differences in self-reported physical fitness in children with different parasitic infections were found are manifold, but do not preclude the use of QoL questionnaires in the elicitation of DWs. Indeed, the questionnaire was particularly useful in assessing physical fitness of those children, who were - supposedly due to parasitic infections - unable to complete the shuttle run test. Hence, we encourage others to use QoL questionnaires to determine not only physical fitness, but also more subtle morbidities.

## Background

The current revision of the Global Burden of Diseases, Injuries and Risk Factors (GBD) 2010 study has sparked new interest in quantifying disability attributable to all kinds of diseases, injuries and risk factors [1]. A crucial variable to estimate the GBD in terms of disability-adjusted life years (DALYs) is the disability weight (DW); a measure which ranges between 0 (perfect health) and 1 (death). Indeed, DWs should capture the disability incurred by an average case suffering from a specific sequela [2]. Of note, DWs are complementary to the utility weights used in the earlier but related quality-adjusted life years (QALYs) metrics [3,4]. In the initial GBD 1990 study, DWs were assessed by person trade-off exercises assigned to panels of public health experts [2]. This dependence on theoretical contemplation was one important source of criticism on the DALY metrics [4-7]. Hence, different research groups started to use one of the growing number of quality of life (QoL) questionnaire tools to estimate patient-based proxies for DWs for a wide range of health impairments [8] - amongst them the often subtle and therefore particularly difficult to elicit morbidity caused by neglected tropical diseases (NTDs). While some results indicate that QoL is significantly lower among individuals affected by NTDs compared to their non-affected counterparts [9,10], others do not [11]. These conflicting findings might reflect the early state of research investigating the relationship between QoL and NTDs, and hence the need for further scientific inquiry has been emphasised [12,13]. However, the ambiguity of this early research also raises questions about the reliability and validity of a questionnaire-based approach in the assessment of impairments in QoL due to NTDs.

We used data from a cross-sectional survey pertaining to schoolchildren's helminth and *Plasmodium *spp. infections, as determined by standardised, quality-controlled parasitological methods. The data were juxtaposed to children's physical fitness, as objectively measured in a maximal multistage 20 m shuttle run test. Additionally, children's self-reported physical fitness was obtained by means of a QoL questionnaire. We determined associations between objectively measured and self-reported physical fitness on one hand, and self-reported physical fitness and infection status on the other hand. Particular emphasis was placed on physical fitness as it is a crucial dimension of QoL. Indeed, physical fitness is included in all generic QoL questionnaires [14-16] and, in our view, the dimension of QoL that can be most easily assessed objectively. We present an evaluation of a QoL questionnaire focusing on the dimension of physical activity. This tool was embedded in a cross-sectional epidemiological survey, which aimed at assessing the effect of helminth and *Plasmodium *spp. infections on schoolchildren's physical fitness in a rural setting of southern Côte d'Ivoire [17].

## Methods

### Study area and data collection

In early 2010, we invited all 204 schoolchildren attending grades 4-6 of Grand Moutcho primary school in Agboville, south Côte d'Ivoire, to participate in a cross-sectional epidemiological survey. In a first step, district health and education authorities, village leaders and teachers were informed about the purpose, procedures and potential risks of the study. After obtaining their oral agreement, written informed consent was sought from the parents or legal guardians of the children, whereas children assented orally.

In a next step, children were asked to fill out a brief questionnaire, assisted by their class teachers if needed. The questionnaire was based on two sections about physical functioning (PF) and physical role (PR) from the most widely used generic SF-36v2 questionnaire (Medical Outcome Trust, Boston, MA, USA; Health Assessment Lab, Boston, MA, USA; QualityMetric, Lincoln, RI, USA) [8,14,18]. Fourteen questions were included and readily adapted to the specific study setting. The questionnaire was pre-tested with support of the head of the school and further revised (see Additional file 1: Questionnaire used to assess self-reported physical fitness in the present study; in French).

Next, participating children were given plastic containers and invited to submit, on the next day, a small portion of their fresh morning stool. After stool collection, from 10:00 hours onwards, children were given a second plastic container and asked to bring a urine sample by 14:00 hours the latest. This procedure was repeated over two consecutive days. Stool and urine samples were transferred to the nearby hospital laboratory of the district town Agboville. Duplicate Kato-Katz thick smears were prepared from each of the two stool samples and quantitatively examined by experienced laboratory technicians for eggs of *Schistosoma mansoni *and soil-transmitted helminths (i.e. *Ascaris lumbricoides*, hookworm and *Trichuris trichiura*) on the same day [19]. Urine samples were subjected to the filtration method and the number of *Schistosoma haematobium *eggs in a filtrate of 10 ml of urine counted under a microscope [20,21]. Ten percent of all parasitological results were re-examined by a senior technician for quality control. In case of disagreement with initial findings, the results were discussed with the respective technician and the corresponding sample re-analysed until agreement was reached.

After the helminthological screening, children were clinically examined by a physician to check their general state of health. Additionally, a rapid diagnostic test (RDT) for malaria was performed (ICT ML01 malaria Pf kit, ICT Diagnostics; Cape Town, South Africa). Children with clinical malaria (defined as positive RDT plus recent history of fever), asthma (assessed by stethoscopy), anaemia (assessed by observing conjunctival vasculature [22]) or dyspnoea (assessed by stethoscopy), according to the physician's appraisal, were excluded from the subsequent fitness test, as participation was considered potentially harmful to them.

Finally, all remaining children were invited to participate in a maximal multistage 20 m shuttle run test to assess the cumulatively covered distance and the aerobe capacity, as measured by their maximal oxygen uptake, the so-called VO_2_max (expressed in ml kg^-1 ^min^-1^) [23,24]. The shuttle run test was conducted in groups of not more than 10 children. The obtained results were used as objectively determined proxies for the children's physical fitness. To ensure that children really tried to reach their maximal physical capacity, their heart rate was observed with a Polar RS400 watch (Polar Electro Europe BV; Zug, Switzerland) and only results of children with more than 180 heart beats per min were considered valid. Throughout the shuttle run test, we monitored ambient air temperature and humidity, as these external factors might influence children's test performance.

### Ethical approval

The study was approved by the institutional research commission of the Swiss Tropical and Public Health Institute (Basel, Switzerland) and received clearance from the ethics committees of Basel (EKBB, reference no. 377/09) and Côte d'Ivoire (reference no. 1993 MSHP/CNER). Insurance coverage was obtained from GNA Assurance (Abidjan, Côte d'Ivoire; policy no. 30105811010001).

At the end of the study, all children attending Grand Moutcho primary school were administered praziquantel (single 40 mg/kg oral dose) and albendazole (single 400 mg oral dose) free of charge, irrespective of their helminth infection status and whether or not they participated in the study. Children who required further medical treatment were referred to the local health service.

### Data analysis

Data were double-entered and cross-checked in Access version 2007 (Microsoft Corporation; Redmond, WA, USA) and analysed in STATA version 10.1 (STATA Corporation; College Station, TX, USA). Questionnaire answers were coded as 1, 2 or 3 (for some questions also 4) with lower scores given to reports of more problems in a certain activity. The individual scores from questions 1 to 10 and 11 to 14, respectively (see Additional file 1: Questionnaire used to assess self-reported physical fitness in the present study) were summed up in order to obtain a summary measure on PF (questions 1 to 10) and PR (questions 11 to 14). While PF is a summary measure for the ability to fulfil distinct physical tasks (e.g. walking, running and climbing), PR pertains to the physical potential to handle certain (social) roles (e.g. learning, helping and playing). According to this procedure, higher values for PF and PR indicate fewer problems in the respective domain. In a last step, scores for PF and PR were transformed to values between 0 and 100, according to equation (1) [25]:(1)

Helminth infection intensities were classified as light, moderate and heavy, using readily available guidelines from the World Health Organization (WHO) [26]. Children's VO_2_max was derived from equation (2), considering age (X_1 _= age in years), the achieved maximal shuttle running speed (X_2 _= speed in km/h) and a linear relation according to Léger and Mercier [23]:(2)

Two different samples were considered in the final analysis in order to assess also the value added by a QoL questionnaire. Sample 1 consisted of all children with complete questionnaire, parasitological and clinical data records. Sample 2 included all children from sample 1 who had not only complete questionnaire, parasitological and clinical data, but also valid physical fitness test results. An attrition analysis was carried out with those children who were included in sample 1, but not in sample 2, i.e. who had complete data records except for the physical fitness test. Besides descriptive statistics, Spearman rank correlation coefficient, uni- and multivariable linear regression models adjusted for participants' age and sex, ambient air temperature and humidity, Fisher's test, χ² and t-test statistics were employed as appropriate to assess statistical significance (p < 0.05).

## Results

### Operational results

**Figure 1:**
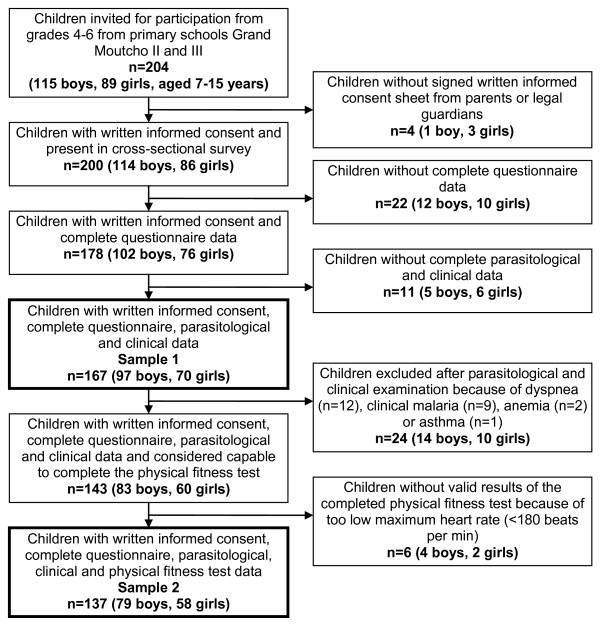
**Flow chart detailing operational study results and the two different samples further considered in the analysis**. The study was carried out in Grand Moutcho school, Agboville, Côte d'Ivoire, in early 2010.

Operational results of the study are summarised in Figure 1. The two final study samples consisted of 167 children (97 boys and 70 girls) and 137 children (79 boys and 58 girls), both with a mean age of 12.0 years (range: 7-15 years). No statistically significant differences in terms of children's sex and age was identified between the two samples (p > 0.05). The 30 children (18 boys and 12 girls) who were part of sample 1 but excluded from sample 2 due to incomplete physical fitness test data had a mean age of 11.8 years (range: 8-14 years). Reasons for having no valid physical fitness test data were the exclusion from the shuttle run test due to medical concerns (i.e. dyspnea (n = 12), clinical malaria (n = 9), anemia (n = 2) and asthma (n = 1)) or reaching too low maximum heart rate (< 180 beats per min) in the shuttle run test (n = 6).

### Parasitological results

Tables 1 and 2 summarise the parasitological results. The prevalence of *S. haematobium, Plasmodium *spp., *S. mansoni*, hookworm and *A. lumbricoides *in the 167 children with complete parasitological results was 84.4%, 74.9%, 54.5%, 14.4% and 1.2%, respectively. No *T. trichiura *infection was diagnosed. High intensity helminth infections were common and only 10.8% of all children were completely helminth-free, while 32.9% harboured a single and 56.3% two or more helminth species concurrently. No significant differences in helminth prevalences, infection intensities or helminth co-infections were found between sample 1 and sample 2 (p > 0.05).

**Table 1:** Parasitic infections and infection intensities in the two samples analysed.

		Sample 1			Sample 2		
		
Parasite	Infection	Male	Female	Total	Male	Female	Total
*S. haematobium*	no	18	8	26	14	7	21
	light (1-49 eggs/10 ml urine)	46	35	81	38	27	65
	heavy (≥ 50 eggs/10 ml urine)	33	27	60	27	24	51
*S. mansoni*	no	52	24	76	42	19	61
	light (1-99 EPG^a^)	31	26	57	26	23	49
	moderate (100-399 EPG^a^)	12	19	31	10	15	25
	heavy (≥ 400 EPG^a^)	2	1	3	1	1	2
*A. lumbricoides*	no	96	69	165	78	57	135
	light (1-4999 EPG^a^)	1	0	1	1	0	1
	moderate (5,000-49,999 EPG^a^)	0	1	1	0	1	1
Hookworm	no	78	65	143	64	53	117
	light (1-1,999 EPG^a^)	18	5	23	15	5	20
	moderate (2,000-3,999 EPG^a^)	1	0	1	0	0	0
*Plasmodium *spp.	no	24	18	42	23	15	38
	yes	73	52	125	56	43	99

^a ^EPG, eggs per gram of stool

Parasitological data stem from a study carried out in Grand Moutcho school, Agboville, Côte d'Ivoire, in early 2010 [17]. Sample 1 with n = 167 observations (97 boys, 70 girls) includes all children with complete questionnaire, parasitological and clinical data. Sample 2 with n = 137 observations (79 boys, 58 girls) includes all children from sample 1 who had not only complete questionnaire, parasitological and clinical data, but also valid shuttle run test results (see also Figure 1). Infection intensities were defined according to WHO guidelines [26].

**Table 2:** Helminth co-infections in the two samples analysed.

	Sample 1			Sample 2		
	
Number of concurrent helminth infections	Male	Female	Total	Male	Female	Total
Zero	13	5	18	11	4	15
One	35	20	55	27	16	43
Two	39	41	80	33	34	67
Three	9	4	13	7	4	11
Four	1	0	1	1	0	1

Parasitological data stem from a study carried out in Grand Moutcho school, Agboville, Côte d'Ivoire, in early 2010 [17]. Sample 1 with n = 167 observations (97 boys, 70 girls) includes all children with complete questionnaire, parasitological and clinical data. Sample 2 with n = 137 observations (79 boys, 58 girls) includes all children from sample 1 who had not only complete questionnaire, parasitological and clinical data, but also valid shuttle run test results (see also Figure 1).

### Results of the questionnaire survey and shuttle run test

Mean scores for PF were 51.3 (95% confidence interval (CI): 48.9-53.6) and 52.2 (95% CI: 49.8-54.7) in samples 1 and 2, respectively (Table 3). Mean scores for PR equalled 50.4 (95% CI: 46.7-54.0) and 51.3 (95% CI: 47.3-55.4). Means of the two objectively measured outcome variables, namely cumulative distance covered by the children in the shuttle run test and VO_2_max, as measured only in sample 2, were 1,301 m (95% CI: 1,242-1,360 m) and 52.7 ml kg^-1 ^min^-1 ^(95% CI: 51.9-53.5 ml kg^-1 ^min^-1^), respectively.

**Table 3:** Summary of the questionnaire scores and shuttle run test results in the two samples analysed.

		Sample 1			Sample 2		
		
Test	Variable	Male	Female	Total	Male	Female	Total
Questionnaire score	Physical functioning	53.0	48.9	51.3	53.2	50.9	52.2
	Physical role	50.9	49.6	50.4	51.2	51.6	51.3
Shuttle run test	Distance	n.a.^a^	n.a.^a^	n.a.^a^	1,452	1,094	1,301
	VO_2_max	n.a.^a^	n.a.^a^	n.a.^a^	54.5	50.3	52.7

^a ^n.a., not applicable

Cumulative distance (in m) and VO_2_max (in ml kg^-1 ^min^-1^) as objectively measured and questionnaire scores on physical functioning and physical role as self-reported variables in samples 1 and 2 from a study carried out in Grand Moutcho school, Agboville, Côte d'Ivoire, in early 2010 [17]. Sample 1 with n = 167 observations (97 boys, 70 girls) includes all children with complete questionnaire, parasitological and clinical data. Sample 2 with n = 137 observations (79 boys, 58 girls) includes all children from sample 1 who had not only complete questionnaire, parasitological and clinical data, but also valid shuttle run test results (see also Figure 1).

Spearman rank correlation coefficients indicated statistically significant and positive correlation between PF and cumulative distance and VO_2_max (Table 4). No such correlation was found between PR and the cumulative distance or VO_2_max.

**Table 4:** Spearman rank correlation coefficients.

	Distance		VO_2_max	
	
	Coefficient	p-value	Coefficient	p-value
Physical functioning	0.215	0.012*	0.186	0.029*
Physical role	0.009	0.922	0.096	0.263

* Statistically significant (p < 0.05)

Cumulative distance (in m) and VO_2_max (in ml kg^-1 ^min^-1^) as objectively measured and questionnaire scores on physical functioning (PF) and physical role (PR) as self-reported variables in sample 2 from a study carried out in Grand Moutcho school, Agboville, Côte d'Ivoire, in early 2010 [17]. Sample 2 with n = 137 observations (79 boys, 58 girls) includes all children with complete questionnaire, parasitological, clinical and shuttle run test results (see also Figure 1).

Uni- and multivariable regression models demonstrated statistically significant correlations of PF and sex with cumulative distance and of PF, sex and age with VO_2_max (Table 5). PR showed no statistically significant association with the cumulative distance or VO_2_max.

**Table 5:** Uni- and multivariable linear regression models.

		Distance			VO_2_max		
		
Model	Explanatories	Coefficient	95% CI^a^	p-value	Coefficient	95% CI^a^	p-value
Univariable	Physical functioning	5.13	1.12, 9.14	0.012*	0.07	0.01, 0.12	0.017*
	Physical role	0.50	-1.95, 2.96	0.685	-0.01	-0.05, 0.02	0.453
	Ambient air temperature	7.25	-11.17, 25.67	0.438	0.03	-0.22, 0.28	0.809
	Ambient air humidity	-2.44	-10.53, 5.66	0.553	0.01	-0.10, 0.12	0.888
	Sex	358.27	255.56, 460.98	< 0.001*	4.23	2.80, 5.66	< 0.001*
	Age	25.14	-8.29, 58.58	0.139	-1.13	-1.53, -0.72	< 0.001*
Multivariable^b,c^	Physical functioning	4.67	0.97, 8.36	0.014*	0.06	0.02, 0.11	0.008*
	Physical role	-0.61	-2.86, 1.63	0.589	-0.01	-0.04, 0.02	0.425
	Ambient air temperature	36.65	-7.34, 80.64	0.102	0.48	-0.06, 1.01	0.080
	Ambient air humidity	10.80	-8.61, 30.21	0.273	0.14	-0.10, 0.37	0.249
	Sex	359.28	257.96, 460.60	< 0.001*	4.36	3.13, 5.60	< 0.001*
	Age	23.51	-5.26, 52.29	0.108	-1.14	-1.49, -0.79	< 0.001*

^a ^95% confidence interval

^b ^Key indicators of the distance regression model: F(6.130) = 10.53, p < 0.001, R^2 ^= 0.327

^c ^Key indicators of the VO_2_max regression model: F(6.130) = 17.26, p < 0.001, R^2 ^= 0.443

* Statistically significant (p < 0.05)

Cumulative distance (in m) and VO_2_max (in ml kg^-1 ^min^-1^) as objectively measured outcome variables and questionnaire scores on physical functioning and physical role as self-reported explanatories in sample 1 from a study carried out in Grand Moutcho school, Agboville, Côte d'Ivoire, in early 2010 [17]. Sample 1 with n = 137 observations (79 boys, 58 girls) includes all children with complete questionnaire, parasitological, clinical and shuttle run test results (see also Figure 1). Ambient air temperature (in°C) and relative humidity (in %) as well as participants sex (reference: female) and age (in years) were included as potential confounders.

Despite diverse infection patterns of the children, uni- and multivariable regression models revealed no statistically significant correlations between participating schoolchildren's score on PF, which proofed to be valid as predictor for their physical fitness, and their parasitic infection status (Table 6).

**Table 6:** Uni- and multivariable linear regression models.

		Sample 1			Sample 2		
		
		Physical functioning			Physical functioning		
		
Model	Explanatories	Coefficient	95% CI^a^	p-value	Coefficient	95% CI^a^	p-value
Univariable	*S. haematobium *light infection	-4.01	-10.84, 2.83	0.249	-0.24	-7.46, 6.97	0.947
	*S. haematobium *heavy infection	-4.47	-11.59, 2.64	0.216	-1.39	-8.84, 6.07	0.713
	*S. mansoni *light infection	-3.22	-8.55, 2.10	0.234	-3.85	-9.33, 1.63	0.167
	*S. mansoni *moderate infection	-2.21	-8.69, 4.26	0.501	1.16	-5.63, 7.94	0.736
	*S. mansoni *heavy infection	3.97	-13.92, 21.85	0.662	-3.44	-23.98, 17.09	0.741
	*A. lumbricoides *light and moderate infection	3.79	-17.81, 25.38	0.730	2.81	-17.59, 23.22	0.785
	Hookworm light and moderate infection	3.15	-3.53, 9.84	0.353	2.08	-4.85, 9.00	0.554
	Concurrent helminth infections: one	-1.71	-9.95, 6.53	0.682	3.22	-5.33, 11.76	0.458
	Concurrent helminth infections: two	-4.73	-12.64, 3.19	0.240	-1.59	-9.73, 6.55	0.699
	Concurrent helminth infections: three and more	-0.95	-11.76, 9.86	0.862	3.75	-7.29, 14.79	0.503
	*Plasmodium *spp. infection	-1.18	-6.60, 4.23	0.667	-1.83	-7.30, 3.62	0.507
	Sex	4.13	-0.59, 8.85	0.086	2.37	-2.57, 7.30	0.345
	Age	-0.13	-1.48, 1.22	0.849	-0.05	-1.44, 1.35	0.948
Multivariable^b,c^	*S. haematobium *light infection	11.24	-34.86, 57.35	0.631	16.50	-27.75, 60.75	0.462
	*S. haematobium *heavy infection	10.88	-35.37, 57.13	0.643	15.96	-28.49, 60.40	0.479
	*S. mansoni *light infection	14.73	-30.72, 60.19	0.523	19.10	-24.10, 62.30	0.383
	*S. mansoni *moderate infection	17.47	-28.19, 63.14	0.451	25.80	-17.61, 69.21	0.242
	*S. mansoni *heavy infection	23.30	-25.63, 72.23	0.348	21.20	-26.58, 68.98	0.382
	*A. lumbricoides *light and moderate infection	12.51	-19.88, 44.91	0.447	11.80	-19.01, 42.61	0.450
	Hookworm light and moderate infection	17.82	-26.46, 62.09	0.428	20.03	-21.82, 61.89	0.345
	Concurrent helminth infections: one	-13.16	-59.73, 33.41	0.577	-13.10	-57.89, 31.70	0.564
	Concurrent helminth infections: two	-31.47	-122.23, 59.28	0.494	-38.70	-125.13, 47.73	0.377
	Concurrent helminth infections: three and more	-46.00	-180.52, 88.52	0.500	-53.39	-181.14, 74.37	0.410
	*Plasmodium *spp. infection	-1.21	-6.94, 4.52	0.678	-2.05	-7.87, 3.76	0.486
	Sex	3.28	-1.82, 8.38	0.206	2.03	-3.25, 7.30	0.448
	Age	0.11	-1.38, 1.60	0.882	-0.01	-1.54, 1.53	0.992

^a ^95% confidence interval

^b ^Key indicators of the regression model based on sample 1: F(13,153) = 0.55, p = 0.891, R^2 ^= 0.045

^c ^Key indicators of the regression model based on sample 2: F(13,123) = 0.66, p = 0.797, R^2 ^= 0.065

* Statistically significant (p < 0.05)

Questionnaire scores on physical functioning as self-reported outcomes and helminth and *Plasmodium *spp. infections (reference: no infection) as parasitologically diagnosed explanatories in samples 1 and 2 from a study carried out in Grand Moutcho school, Agboville, Côte d'Ivoire, in early 2010 [17]. Sample 1 with n = 167 observations (97 boys, 70 girls) includes all children with complete questionnaire, parasitological and clinical data. Sample 2 with n = 137 observations (79 boys, 58 girls) includes all children from sample 1 who had not only complete questionnaire, parasitological and clinical data, but also valid shuttle run test results (see also Figure 1). Infection intensities were defined according to WHO guidelines [26]. Categories of explanatories with n ≤ 1 observations were combined with the next best category of the same explanatory. Participants sex (reference: female) and age (in years) were included as potential confounders.

### Results of the attrition analysis

An attrition analysis of the group of 30 children excluded in sample 2 showed that this group was neither significantly different in terms of their sex- or age-composition, nor their respective parasite prevalences, infection intensities or levels of multiple helminth species infections (p > 0.05). However, as expected because of their medical complaints, they reported lower PF scores as a group (mean = 46.8; 95% CI: 39.9-53.8). Furthermore, while the three completely parasite-free children achieved considerably higher mean PF scores (mean = 66.7; 95% CI: 16.5-116.9), the 27 parasitized children reported lower mean PF scores (mean = 44.6; 95% CI: 37.8-51.5) than their peers from sample 2 (Table 7).

**Table 7:** Attrition analysis of mean questionnaire scores on self-reported physical functioning (PF).

	Sample 2			Excluded		
	
Parasitic infection status	n	mean score PF	95% CI^a^	n	mean score PF	95% CI^a^
Parasite-free	15	51.7	41.3, 62.1	3	66.7	16.5, 116.9
Parasitized	122	52.3	49.8, 54.8	27	44.6	37.8, 51.5
All	137	52.2	49.7, 54.7	30	46.8	39.9, 53.8

^a ^95% confidence interval

Questionnaire scores and parasitological results stem from a study carried out in Grand Moutcho school, Agboville, Côte d'Ivoire, in early 2010 [17]. Sample 2 with n = 137 observations (79 boys, 58 girls) includes all children who had complete questionnaire, parasitological, clinical and also shuttle run test results (see also Figure 1). The group of excluded with n = 30 observations (18 boys, 12 girls) consists of all children with complete questionnaire, parasitological and clinical data, but no valid shuttle run test results (see also Figure 1). Together, the two groups add up to sample 1 with n = 167 observations (97 boys, 70 girls), which includes all children with complete questionnaire, parasitological and clinical data, but not necessarily valid shuttle run test results.

## Discussion

To our knowledge, this is one of the first attempts to compare objectively measured and self-reported physical fitness in an area where malaria, schistosomiasis and soil-transmitted helminthiasis co-exist [17]. We found a significant association between the cumulative distance covered in a maximal multistage 20 m shuttle run test by 7-15 year old schoolchildren and the index PF, as calculated from children's answers to 10 questions in a simplified QoL questionnaire. The association between PF and the children's estimated VO_2_max was also statistically significant, regardless of whether uni- or multivariable linear regression analyses were performed. VO_2_max is a widely used proxy for the aerobe capacity, and hence a key indicator for the physical performance of a person [24]. In contrast, the index PR, as calculated from the children's answers on four additional questions in the same simplified QoL questionnaire, was neither associated with cumulative distance nor VO_2_max.

The findings that (i) PF was a better predictor than PR for objectively measured physical fitness in the present study and that (ii) the association between PF and cumulative distance was even better than the association between PF and VO_2_max are reasonable. These claims are justified as follows. First, PF literally includes questions about fulfilling distinct physical functions such as running (questions 1 and 2), climbing a hill (questions 4 and 5) or walking (questions 7-9), while PR is more concerned with the physical ability to fulfil certain (social) roles (questions 11-14). Second, three questions (questions 7-9) are directly asking about the ability to cover certain distances (see also Additional file 1: Questionnaire used to assess self-reported physical fitness in the present study).

Interestingly, no association between parasitic infection status and the indices based on self-reported physical fitness was identified. However, by logical deduction, this finding had to be expected after no association was detected between parasitic infections and the objectively measured physical fitness in the umbrella study into which the current investigation was embedded [17] and a strong association was found between the objectively measured physical fitness and self-reported physical fitness in the present study. The finding that the parasitic infection status does not prejudice physical fitness is also in line with some older investigations [27,28], but contradicts newer research [29-31] and the widely held belief that schistosomiasis, soil-transmitted helminthiasis and malaria impair the infected individuals' physical fitness. Some potential explanations and approaches for further research to better understand and solve this discrepancy have been discussed elsewhere [17]. In brief, the participating Ivorian schoolchildren presented with clearly better physical fitness as for example age-matched Canadian counterparts [23]. Differences in life-styles and nutrition might, at least to some degree, explain this observation [32-35]. It has also been implied that the Ivorian children with a parasitic infection could maybe mask the incurred disability by their generally excellent level of physical fitness. The children may adapt quite well to parasitic infections that are acquired early in their life and the children do not yet experience disability caused by advanced chronic disease. Furthermore, it has been suggested that children attending school might not be fully representative in a given epidemiological setting, as children from poorest and furthest away households are less likely to be registered at school and at the same time more likely to be infected with parasites. It is also conceivable that children really suffering from the adverse effects of the infections rest at home because of their signs and symptoms, and hence did not take part in our study.

Of particular interest was the fact that the use of a QoL questionnaire, which proofed to be a valid tool to assess the schoolchildren's physical fitness in the respective study area, permitted the inclusion of an additional 30 children who had to be excluded from the shuttle run test due to medical concerns. An attrition analysis indicated that this excluded group of children was not different from the group that could successfully complete the shuttle run test with regard to sex, age or parasitic infection status. However, the mean PF scores of the parasitized and excluded children was considerably lower than the mean PF scores of the children who were parasitized as well but able to participate in the shuttle run test. It could be hypothesized that mainly children who suffer the most from their parasitic infections were excluded from the physical fitness test - precisely because of experiencing severe sequelae. Supposedly due to the relatively small sample size, which was at least partially owed to our rigorous study design, the difference in PF between the two groups showed no statistical significance. As revealed by the analysis of the two different samples in Table 6, the inclusion of the 30 children who were probably most seriously affected by parasitic infections could not overpower the results of the other 137 children. Nevertheless, their inclusion was only possibly thanks to the QoL questionnaire results and their exclusion would have led most likely to biased results.

## Conclusion

We consider the questionnaire employed in the present study as a valid tool to assess schoolchildren's physical fitness in the respective study area. Nevertheless, further validation in other settings of Côte d'Ivoire (e.g. urban areas) and elsewhere in sub-Saharan Africa is warranted. The questionnaire was particularly useful in assessing physical fitness of those children who were unable to complete an exhausting physical fitness test. Future and preferably larger studies to assess disability caused by helminthic infections, other NTDs and malaria should use a test-retest design with intermittent treatment or even piggyback on continuous preventive chemotherapy campaigns. Furthermore, they should also consider dimensions of QoL other than physical fitness, for instance bodily pain and potentially following affection of vitality, mental health and social functioning. However, these dimensions are exceedingly difficult to measure with tools that do not include one or the other form of a questionnaire. This is even more true when affection is subtle as it is often the case with NTDs. We believe that QoL questionnaires will gain further importance in eliciting and quantifying disability caused by NTDs. Hence, further development and validation of such tools, for instance by using mixed methods triangulation approaches, is warranted.

## Competing interests

The authors declare that they have no competing interests.

## Authors' contributions

TF, IM, JU and EKN designed the study; IM, JTC and AKY implemented the study; TF and IM managed, analysed and interpreted the data; TF, IM and JU wrote the paper. JU and EKN supervised the different phases of the study. All authors read, revised and approved the final manuscript.

## Supplementary Material

Additional file 1 **Questionnaire employed to assess self-reported physical fitness in the present study**. Two sections about physical functioning and physical role from the widely used SF-36v2 questionnaire (Medical Outcome Trust, Boston, MA, USA; Health Assessment Lab, Boston, MA, USA; QualityMetric, Lincoln,, RI, USA) [14,18] were used as templates, adapted to the specific study setting, pre-tested and further revised. Click here for file

## References

[B1] Global burden of disease study http://www.globalburden.org/accessed: 27 Jan 2011

[B2] Murray CJL Murray CJL, Lopez AD Rethinking DALYs The Global Burden of Disease: A Comprehensive Assessment of Mortality and Disability from Diseases, Injuries, and Risk Factors in 1990 and Projected to 2020 1996 I Cambridge: Harvard University Press 1 99

[B3] Gold MR Stevenson D Fryback DG HALYs and QALYs and DALYs, Oh My: similarities and differences in summary measures of population health Annu Rev Public Health 2002 23 115 134 10.1146/annurev.publhealth.23.100901.140513 11910057

[B4] Arnesen T Nord E The value of DALY life: problems with ethics and validity of disability adjusted life years BMJ 1999 319 1423 1425 10574867 10.1136/bmj.319.7222.1423 PMC1117148

[B5] King CH Bertino AM Asymmetries of poverty: why global burden of disease valuations underestimate the burden of neglected tropical diseases PLoS Negl Trop Dis 2008 2 e209 10.1371/journal.pntd.0000209 18365036 PMC2267491

[B6] Anand S Hanson K Disability-adjusted life years: a critical review J Health Econ 1997 16 685 702 10.1016/S0167-6296(97)00005-2 10176779

[B7] Reidpath DD Allotey PA Kouame A Cummins RA Measuring health in a vacuum: examining the disability weight of the DALY Health Policy Plan 2003 18 351 356 10.1093/heapol/czg043 14654511

[B8] Garratt A Schmidt L Mackintosh A Fitzpatrick R Quality of life measurement: bibliographic study of patient assessed health outcome measures BMJ 2002 324 1417 1421 10.1136/bmj.324.7351.1417 12065262 PMC115850

[B9] Jia TW Zhou XN Wang XH Utzinger J Steinmann P Wu XH Assessment of the age-specific disability weight of chronic schistosomiasis japonica Bull World Health Organ 2007 85 458 465 10.2471/BLT.06.033035 17639243 PMC2636356

[B10] Jia TW Utzinger J Deng Y Yang K Li YY Zhu JH King CH Zhou XN Quantifying quality of life and disability of patients with advanced schistosomiasis japonica PLoS Negl Trop Dis 2011 5 e966 10.1371/journal.pntd.0000966 21358814 PMC3039691

[B11] Ziegelbauer K Steinmann P Zhou H Du ZW Jiang JY Fürst T Jia TW Zhou XN Utzinger J Self-rated quality of life and school performance in relation to helminth infections: case study from Yunnan, People's Republic of China Parasit Vectors 2010 3 61 10.1186/1756-3305-3-61 20650011 PMC2923135

[B12] King CH Dangerfield-Cha M The unacknowledged impact of chronic schistosomiasis Chronic Illn 2008 4 65 79 10.1177/1742395307084407 18322031

[B13] Schistosomiasis Consortium for Operational Research and Evaluation (SCORE): Subtle morbidity cohort studies http://score.uga.edu/Subtle.Morbidity.html accessed: 27 Jan 2011

[B14] SF-36v2 health survey http://www.sf-36.org/tools/pdf/SF-36v2_Standard_Sample.pdf accessed: 27 Jan 2011

[B15] EQ-5D-5L http://www.euroqol.org/fileadmin/user_upload/Documenten/PDF/Languages/Sample_UK__English__EQ-5D-5L.pdf accessed: 27 Jan 2011

[B16] The World Health Organization Quality of Life (WHOQOL)-bref http://www.who.int/substance_abuse/research_tools/en/english_whoqol.pdf accessed: 27 Jan 2011 10.1007/s11136-004-5327-1 15892436

[B17] Müller I Coulibaly JT Fürst T Knopp S Hattendorf J Krauth SJ Stete K Righetti AA Glinz D Yao AK Effect of schistosomiasis and soil-transmitted helminth infections on physical fitness of school children in Côte d'Ivoire PLoS Negl Trop Dis 2011 5 e1239 10.1371/journal.pntd.0001239 PMC3139653 21811643

[B18] SF-36v2 health survey http://www.qualitymetric.com/WhatWeDo/GenericHealthSurveys/SF36v2HealthSurvey/tabid/185/Default.aspx accessed: 27 Jan 2011

[B19] Katz N Chaves A Pellegrino J A simple device for quantitative stool thick-smear technique in schistosomiasis mansoni Rev Inst Med Trop São Paulo 1972 14 397 400 4675644

[B20] World Health Organization Helminth Control in School-aged Children: A Guide for Managers of Control Programmes 2002 Geneva: WHO

[B21] Savioli L Hatz C Dixon H Kisumku UM Mott KE Control of morbidity due to *Schistosoma haematobium *on Pemba Island: egg excretion and hematuria as indicators of infection Am J Trop Med Hyg 1990 43 289 295 2121056 10.4269/ajtmh.1990.43.289

[B22] Kent AR Elsing SH Hebert RL Conjunctival vasculature in the assessment of anemia Ophthalmology 2000 107 274 277 10.1016/S0161-6420(99)00048-2 10690824

[B23] Léger LA Mercier D Gadoury C Lambert J The multistage 20 metre shuttle run test for aerobic fitness J Sports Sci 1988 6 93 101 3184250 10.1080/02640418808729800

[B24] American College of Sports Medicine ACSM's Guidelines for Exercise Testing and Prescription 2008 Philadelphia: Lippincott Williams & Wilkins21694556

[B25] Ware JE Kosinski M Turner-Bowker DM Gandek B User's Manual for the SF-12v2 Health Survey 2007 Lincoln: QualityMetric21706089

[B26] World Health Organization Prevention and control of schistosomiasis and soil-transmitted helminthiasis: report of a WHO expert committee WHO Tech Rep Ser 2002 912 1 57 12592987

[B27] Davies CT The effects of schistosomiasis, anaemia and malnutrition on the responses to exercise in African children J Physiol 1973 230 Suppl 27 4702431

[B28] Walker AR Walker BF Richardson BD Smit PJ Running performance in South African Bantu children with schistosomiasis Trop Geogr Med 1972 24 347 352 4648648

[B29] Kvalsvig JD The effects of schistosomiasis haematobium on the activity of school children J Trop Med Hyg 1986 89 85 90 3095562

[B30] Ndamba J Makaza N Munjoma M Gomo E Kaondera KC The physical fitness and work performance of agricultural workers infected with *Schistosoma mansoni *in Zimbabwe Ann Trop Med Parasitol 1993 87 553 561 8122916 10.1080/00034983.1993.11812810

[B31] King CH Dickman K Tisch DJ Reassessment of the cost of chronic helmintic infection: a meta-analysis of disability-related outcomes in endemic schistosomiasis Lancet 2005 365 1561 1569 10.1016/S0140-6736(05)66457-4 15866310

[B32] Brown T Bell M Off the couch and on the move: global public health and the medicalisation of nature Soc Sci Med 2007 64 1343 1354 10.1016/j.socscimed.2006.11.020 17188788

[B33] Mitchikpe CE Dossa RA Ategbo EA Van Raaij JM Kok FJ Seasonal variation in food pattern but not in energy and nutrient intakes of rural Beninese school-aged children Public Health Nutr 2009 12 414 422 18616847 10.1017/S1368980008002929

[B34] Choi BC Hunter DJ Tsou W Sainsbury P Diseases of comfort: primary cause of death in the 22nd century J Epidemiol Community Health 2005 59 1030 1034 10.1136/jech.2005.032805 16286489 PMC1732964

[B35] Ezzati M Lopez AD Rodgers A Vander Hoorn S Murray CJL Selected major risk factors and global and regional burden of disease Lancet 2002 360 1347 1360 10.1016/S0140-6736(02)11403-6 12423980

