# Supplementary material for: 6Questionnaire-based approach to assess schoolchildren's physical fitness and its potential role in exploring the putative impact of helminth and Plasmodium spp. infections in Côte d'Ivoire
Source: Parasit Vectors. 2011 Jun 24;4:116. doi: 10.1186/1756-3305-4-116 (PMC3157450; doi:10.1186/1756-3305-4-116)
Supplement: Additional file 1 — Questionnaire employed to assess self-reported physical fitness in the present study. Two sections about physical functioning and physical role from the widely used SF-36v2 questionnaire (Medical Outcome Trust, Boston, MA, USA; Health Assessment Lab, Boston, MA, USA; QualityMetric, Lincoln,, RI, USA) [14,18] were used as templates, adapted to the specific study setting, pre-tested and further revised. [file 1756-3305-4-116-S1.PDF]

Cher élève, chère élève,

Nous avons rédigé 14 brèves questions concernant ton avis sur ta forme physique. Ce n'est pas un test avec des bonnes ou des fausses réponses. Pour nous, il est plus important d'apprendre comment tu vas et c'est seulement ton honnête opinion qui est importante. Alors, nous te prions de remplir le questionnaire suivant complètement.

1. Pour commencer, lis chaque question.
2. Assure toi que tu comprends toutes les questions. Si tu n'es pas sûr(e), tu peux toujours poser des questions à l'équipe ou à l'instituteur.
3. Contrôle toutes les réponses données, choisis la réponse qui est la plus vraie pour ta situation et puis marque le box correspondant □ avec une croix.
4. Choisis seulement une réponse par question.

Tout d'abord, nous avons besoin de ton nom.

Ecris ton nom ici: ____

Tu trouves en suivant les 14 brèves questions concernant ton avis sur ta forme physique. Chaque question décrit les activités quotidiennes au cours desquelles tu peux rencontrer des problèmes comme l'essoufflement, une fatigue ou d'épuisement à cause de ta forme physique. Maintenant c'est à toi de choisir la réponse qui est la plus vraie pour ta situation.

1) *As-tu des problèmes pour faire des activités très fatigantes, comme par exemple courir très vite, soulever des choses très lourdes ou jouer au foot sans arrêter?*
□ Oui, beaucoup de problèmes. □ Oui, quelques problèmes. □ Non, pas de problème.

2) *As-tu des problèmes de faire des activités moyennement fatigantes, comme par exemple courir lentement, bouger une table ou jouer à cache-cache?*
□ Oui, beaucoup de problèmes. □ Oui, quelques problèmes. □ Non, pas de problème.

3) *As-tu des problèmes pour soulever ou porter ton sac à dos ou sac de riz de 5 kilogramme?*
□ Oui, beaucoup de problèmes. □ Oui, quelques problèmes. □ Non, pas de problème.

4) *As-tu des problèmes pour monter brièvement sur une colline basse?*
□ Oui, beaucoup de problèmes. □ Oui, quelques problèmes. □ Non, pas de problème.

5) *As-tu des problèmes pour monter sur une colline raide pendant un temps prolongé?*
□ Oui, beaucoup de problèmes. □ Oui, quelques problèmes. □ Non, pas de problème.

6) *As-tu des problèmes pour te pencher en avant, te baisser ou t'agenouiller?*
□ Oui, beaucoup de problèmes. □ Oui, quelques problèmes. □ Non, pas de problème.

7) *As-tu des problèmes pour aller à pied de l'école de Moutcho jusqu'au marché de Moutcho (environs 100 mètres)?*
□ Oui, beaucoup de problèmes. □ Oui, quelques problèmes. □ Non, pas de problème.

8) *As-tu des problèmes pour aller à pied de l'école de Moutcho jusqu'au bout de village Moutcho (environs 400 - 600 mètres)?*
□ Oui, beaucoup de problèmes. □ Oui, quelques problèmes. □ Non, pas de problème.

9) *As-tu des problèmes pour aller à pied de l'école de Moutcho jusqu' à l'école Nakoi (environs 1'000 mètres)?*
□ Oui, beaucoup de problèmes. □ Oui, quelques problèmes. □ Non, pas de problème.

10) *As-tu des problèmes pour te baigner, te laver ou t'habiller?*
□ Oui, beaucoup de problèmes. □ Oui, quelques problèmes. □ Non, pas de problème.

11) *As-tu eu des problèmes pour apprendre tes leçons, aider quelqu'un de ta famille ou jouer avec tes amis aussi long temps que d'habitude à cause de ta forme physique pendant le dernier mois?*
□ Oui, toujours. □ Oui, souvent. □ Oui, mais rarement. □ Non, jamais.

12) *As-tu eu des problèmes à faire la même quantité de devoirs à l'école ou à la maison comme d'habitude à cause de ta forme physique pendant le dernier mois?*
□ Oui, toujours. □ Oui, souvent. □ Oui, mais rarement. □ Non, jamais.

13) *Pouvais-tu seulement faire certaines activités quotidiennes (comme apprendre pour l'école, aider quelqu'un de ta famille ou jouer) mais pas toutes à cause de ta forme physique pendant le dernier mois?*
□ Oui, toujours. □ Oui, souvent. □ Oui, mais rarement. □ Non, jamais.

14) *As-tu eu besoin de faire plus d'effort que d'habitude pour apprendre tes leçons, aider quelqu'un de ta famille ou jouer avec tes amis à cause de ta forme physique pendant le dernier mois?*

□ Oui, toujours. □ Oui, souvent. □ Oui, mais rarement. □ Non, jamais.

Et voilà, c'est déjà fini. Merci beaucoup pour ta merveilleuse collaboration.

☺
